# Viscoelastic Biomarkers of Ex Vivo Liver Samples via Torsional Wave Elastography

**DOI:** 10.3390/diagnostics10020111

**Published:** 2020-02-19

**Authors:** Inas H. Faris, Juan Melchor, Antonio Callejas, Jorge Torres, Guillermo Rus

**Affiliations:** 1 Department of Structural Mechanics, University of Granada, 18071 Granada, Spain; inas@ugr.es (I.H.F.); geresez@ugr.es (J.T.); grus@ugr.es (G.R.); 2 Instituto de Investigación Biosanitaria, ibs.GRANADA, 18012 Granada, Spain; jmelchor@ugr.es; 3 Department of Statistics and Operations Research, University of Granada, 18071 Granada, Spain; 4 Excellence Research Unit “ModelingNature” (MNat), Universidad de Granada, 18071 Granada, Spain

**Keywords:** Shear Wave Elastography Imaging, Torsional Wave Elastography, mechanical biomarkers, tissue biomarkers, Kelvin–Voigt viscoelasticity

## Abstract

The clinical ultrasound community demands mechanisms to obtain the viscoelastic biomarkers of soft tissue in order to quantify the tissue condition and to be able to track its consistency. Torsional Wave Elastography (TWE) is an emerging technique proposed for interrogating soft tissue mechanical viscoelastic constants. Torsional waves are a particular configuration of shear waves, which propagate asymmetrically in-depth and are radially transmitted by a disc and received by a ring. This configuration is shown to be particularly efficient in minimizing spurious *p-waves* components and is sensitive to mechanical constants, especially in cylinder-shaped organs. The objective of this work was to validate (TWE) technique against Shear Wave Elasticity Imaging (SWEI) technique through the determination of shear wave velocity, shear moduli, and viscosity of ex vivo chicken liver samples and tissue mimicking hydrogel phantoms. The results of shear moduli for ex vivo liver tissue vary 1.69–4.0kPa using TWE technique and 1.32–4.48kPa using SWEI technique for a range of frequencies from 200 to 800Hz. Kelvin–Voigt viscoelastic parameters reported values of μ = 1.51kPa and η = 0.54Pa·s using TWE and μ = 1.02kPa and η = 0.63Pa·s using SWEI. Preliminary results show that the proposed technique successfully allows reconstructing shear wave velocity, shear moduli, and viscosity mechanical biomarkers from the propagated torsional wave, establishing a proof of principle and warranting further studies.

## 1. Introduction

Ultrasonography has been widely used for diagnosis since it was first used in clinical applications in the 1970s [1]. Since then, new ultrasound modalities have been developed to provide information for a better diagnosis. Elastography enables us to determine the viscoelastic properties of living tissues and has been used since the 1990s in a growing number of medical applications [2]. It reproduces and replaces the subjective palpation done by clinicians [3] and can reach to deep organs that can not be palpated by hands.

Mapping the stiffness can be estimated from static methods, i.e., the analysis of the strain in the tissue under a pressure, or by dynamic methods, i.e. the propagation of shear waves through the tissue [4]. Dynamic elastography (DE) techniques are based on applying a force that deforms the tissue and tracking tissue deformation due to this force. The latter elastography techniques provided clinicians with quantitative mechanical biomarkers compared to static methods [5]. However, they need complex systems able to generate the shear waves either by a mechanical vibrator, through acoustic radiation force (ARF), or by harmonic vibration, in the case of Magnetic Resonance Elastography (MRE), and to image or quantify the small displacements induced by the shear wave [6,7,8,9].

Chronic liver diseases are an important public health problem. It is estimated that more than two million people die each year due to chronic liver diseases [10]. For this, mechanisms for its prevention, correct detection, and treatment are demanded [11].

Producing adequate shear waves in soft tissue is not straightforward due to the diffractions and reflections. Despite this, shear wave elastography (SWE) and in parallel MRE have been used to evaluate the staging of liver fibrosis [12,13,14,15,16] among a wide range of pathologies. In this study, comparisons of the proposed technology were done against SWE.

Torsional waves are shear elastic waves that propagate through soft tissue radially and in-depth in a curved geometry. Application of torsional waves to sense soft tissue architecture has been proved to enable a new class of biomarkers that quantify the mechanical functionality of any soft tissue [17]. Abnormalities in the structural architecture of soft tissues are intimately linked to a broad range of pathologies including solid tumors, atherosclerosis, liver fibrosis, and osteoarticular syndromes [8]. The unexplored nature and applicability span of these mechanical biomarkers and torsional waves provides a very interesting diagnostic technology. The need for comparative and repetitive studies is clear. Validation studies are demanded due to the increased interest in the viscoelastic parameters obtained from elastography techniques [18].

In this study, the generation and detection of torsional waves through the proposed technology (TWE) developed by our group [19,20] was used to obtain mechanical biomarkers in terms of shear wave velocity and shear moduli of ex vivo soft tissue. Liver samples were chosen in this work considering that the assessment of elastic biomarkers in abdominal tissue has been widely analyzed by SWE since the beginnings of the technique. Consequently, there are numerous studies in the literature to validate against.

Since TWE is an emerging technology, our objective was to compare scans of ex vivo liver samples with ones obtained from dynamic elastography techniques. Thus far, there is not enough scientific evidence in the literature to validate the most recent technologies. The first attempt to validate TWE was made by Callejas et al. [21] using classical rheometry. In this study, the validation was made using a Verasonics Vantage system (Verasonics, Inc., Kirkland, WA, USA). Shear waves were generated by an Acoustic Radiation Force Impulse (ARFI) to reduce some limitations of the previous work since classical rheometry works in a much lower frequency range than TWE. One important contribution of this work with respect to the prior is being able to compare the results of both techniques in the same frequency range. A second approach was done, in which a reconstruction of the Kelvin–Voigt (KV) viscoelastic parameters was performed using a Probabilistic Inverse Problem (PIP) approach in tissue mimicking hydrogel phantoms; the results obtained from the TWE technique were compared against the synthetic signals from a Finite Difference Time Domain (FDTD) [22]. The aim was to validate the efficacy of the proposed reconstruction method via PIP. However, in this work, the reconstruction was done using the Time of Flight (TOF); Kelvin–Voigt (KV) and Maxwell (M) viscoelastic parameters were compared for soft tissue instead of just tissue mimicking hydrogel phantoms.

## 2. Materials and Methods

**Figure 1:**
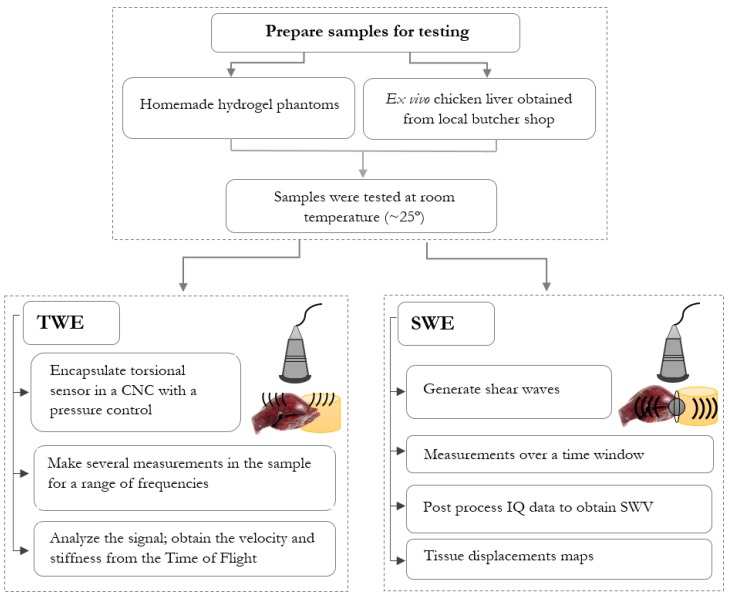
Flow chart showing the steps for scans performed with both techniques, Torsional Wave Elastography (TWE) and Shear Wave Elastography Imaging (SWEI).

Two techniques were used to obtain mechanical elastic and viscoelastic biomarkers. The first, developed by our group, was TWE, and the other technique used a commercially available system for shear wave generation, a Verasonics Vantage system. The elasticity measurements using 2D SWEI and TWE may be expressed as either shear wave velocity (m/s) or shear moduli (kPa). The procedure for the samples scan is shown in Figure 1. Basically, it consists of preparing the samples (ex vivo liver and hydrogel phantoms) for testing; measuring them by generating torsional and shear waves; capturing the propagation of these waves; and, finally. analyzing the signal to reconstruct the biomechanical markers.

### 2.1. Hydrogel Phantoms and Ex Vivo Samples

To assess the properties and feasibility of SWEI in a safe and repeatable manner, it is necessary to use phantoms with ultrasonic properties mimicking those of soft tissue, eliminating real tissue heterogeneity, anisotropy, and variability; thus, hydrogel phantoms were prepared. Three fresh ex vivo samples of chicken liver were also tested for testing real scenarios. To perform ultrasound stiffness imaging, ex vivo samples were kept at room temperature at 25° before testings. Each sample was scanned three times in two different regions.

**Figure 2:**
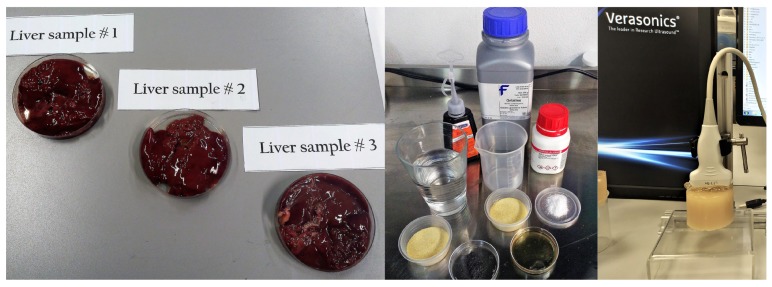
Three ex vivo liver samples, phantom ingredients, and one of the phantoms subjected to shear wave elastography imaging.

To compare and determine the effect of the viscosity, two homemade homogeneous phantoms were prepared. Both were made from porcine gelatin powder at 7.5% gelatin concentration (Fisher Chemical, Leicestershire, UK) and 0.5% of sodium dodecyl sulfate (Sigma-Aldrich Corp., St. Louis, MO, USA). To capture the shear wave, we used enhancements for the ultrasound imaging 1% of castor oil in one phantom and 0.5% graphite particles in the second one. The manufacturing process of the phantoms followed the standard used in the literature and particularly the methodology proposed by Park et al. [23] and Dunmire et al. [24]. Figure 2 shows ex vivo liver samples used for this work, the ingredients for phantom manufacturing, and one of the hydrogel phantoms being scanned.

### 2.2. Torsional Wave Elastography

The torsional wave sensor is based on a novel arrangement of concentric sandwiches of piezo- and electromechanical elements. The emitter transmitting the waves consists of a PLA (polylactic acid) disk, printed in 3D, whose rotational movement is due to an electromechanical actuator. The receiver is formed by two PLA rings with four slots in the inner face of the ring, where the four ceramic piezoelectric elements are fitted [25,26,27]. This allows the precise interrogation of soft tissue mechanical functionality in cylindrical geometries. Dealing with this type of geometry is a challenge for current elastography approaches in small organs.

**Figure 3:**
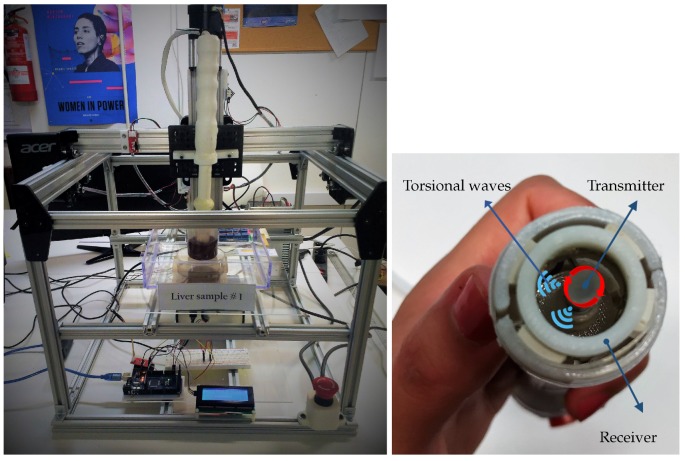
Set-up for measurements using TWE technique. The picture was taken during the measurements at the Ultrasonics Lab at the University of Granada. The figure on the left is a computer numerical control (CNC) system for positioning and pressure-control of the TWE probe. The right figure shows a cross-section of the TWE probe.

Figure 3 shows the TWE probe developed by our group. The left sub-figure shows the sensor encapsulated in a CNC (computer numerical control) system that allows measuring within an exact position and at the same time exerting a controlled pressure on the sample. The right sub-figure shows a cross-section of the TWE probe. More details of the probe can be found in the work of Callejas et al. [21].

#### Time of Flight (TOF)- Signal Processing

Physically, torsional waves are originated by the actuator (transmitter, right part of Figure 3) and are transmitted through the specimens to the piezoelectric sensor, where they produce the deformation thereof and, consequently, an electric potential catchable by an oscilloscope.

To compensate for the mechanical and electronic crosstalk, a measurement is first taken in air, without contact with the specimen, which generates a signal transmitted mechanically inside the probe and electronically in air, under similar humidity conditions. This signal is stored and subtracted from the signals on the specimens, effectively compensating for the mechanical and electronic crosstalk. This signal is averaged 10 times for noise reduction, using a repetition rate that allows full dissipation of preceding waves. The total time of measurement is a quarter of a second, which is enough to register the desired frequency.

**Figure 4:**
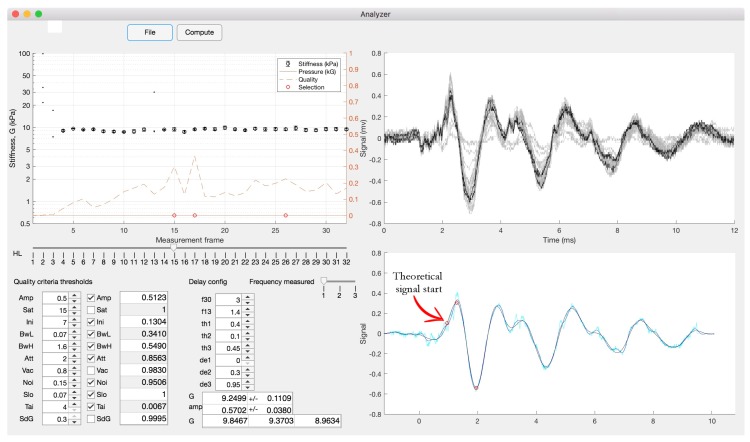
Example of an output of the analyzer software used to analyze the signals obtained from the TWE technique. The upper left sub-figure shows the stiffness obtained at each measurement frame. The lower-right sub-figure shows the theoretical signal start.

The remaining signal has traveled across the specimen and also through some mechanical parts of the probe. The apparent TOF is estimated from the subtracted signal as above, and after using a low pass filter at three times the center frequency, in three complementary ways: (1) by estimating the time where the signal amplitude surpasses 30% of the max level; (2) by finding the first peak after that threshold; and (3) by finding the next negative peak, as indicated in Figure 4. The time of the theoretical signal start is estimated by subtracting the corresponding fractions of the period corresponding to the excitation frequency.

The apparent time of flight (TOF) is, therefore, the sum of the TOF within the specimen plus the TOF across the components of the probe, which is called internal delay. The latter is a probe-specific constant that needs to be calibrated against SWEI and subtracted prior to computing the speed by dividing the distance by TOF within the specimen (Equation (1)), yielding Equation (2). Acquisition parameters for TWE technique used for both ex vivo liver samples and tissue-mimicking hydrogel are shown in Table 1.

(1) $$c_{s}\;=\;\frac{\mathrm{distance}}{\mathrm{TOF}}$$

(2) $$c_{s}\;=\;\frac{\mathrm{distance}}{\mathrm{TOF}-\mathrm{delay}}$$

### 2.3. Shear Wave Elastography

Acoustic radiation force imaging (ARFI) was introduced by Nightingale et al. [28]. This method uses focused ultrasound to generate localized displacement of a few microns via an ARF impulse within the tissue. During the impulse, the acoustic wave propagates through the tissue. Local displacements are related to the mechanical properties of the tissue, which deforms in response to the focused ARF excitation, thus shear waves propagate away from it [29]. Finally, the displacement generated by the ARF is then mapped, within the focal region of each push within a specified region of interest (ROI) at a known time after stopping the push. The tissue displacement response within the region of the push is directly related to the magnitude of the applied force and inversely related to the tissue stiffness [8,28].

Shear wave elastography as ARFI also uses an ARF to excite the medium and generate shear waves and produces a quantitative elasticity map of the medium in real-time. The technique can be subdivided into the creation of the Mach-cone, where ultrasound beams are focused successively at different depths to create spherical waves at each focal point. The different generated spherical waves interfere constructively along a Mach-cone creating two quasi-plane shear wavefronts propagating in opposite directions in the imaging plane [7].

**Figure 5:**
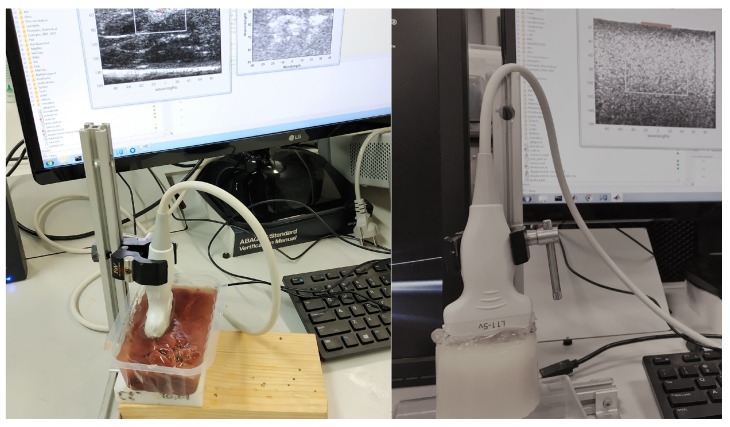
Set-up for measurements using SWEI. The picture was taken during the measurements at the Ultrasonics Lab at the University of Granada. In the left image, the ex vivo liver sample is measured while one of the hydrogel phantoms is shown in the right image.

The Verasonics Vantage US research system is used to generate the push sequences and generate the shear waves. Verasonics is compatible with many transducers and offers big flexibility in sequence design. Additionally, Verasonics provides direct access to the raw channel data from each element of the array, as well as a software beamformer to form ultrasound images [30]. In Figure 5, one can see the Vantage Verasonics system during measurements and the Verasonics L11−5v transducer used in this work is shown.

The Verasonics vantage 128 system was used to perform the SWEI. The system uses the MATLAB programming environment to create the protocol of measurements of SWEI. The sequence of steps is as follows: the programmer writes a programming script to generate an imaging sequence, which generates a collection of objects that are loaded into the Verasonics scanner during runtime. The main parameters for the script are: (1) the push and track transmit frequencies; (2) the push duration; (3) the push and track transmit aperture; (4) the sampling frequencies; and (5) the pulse repetition interval. Details and sequences of the Verasonics script can be found in the work of Deng et al. [30]. In this study, a different transducer was used. Properties of the L11−5v 128 elements linear array transducer are shown in Table 2.

Table 3 lists the SWEI acquisition parameters used in this study. The push transmit frequency was set to the center of the transducer to allow maximum transmission efficiency to transfer the ARFI to the tissue. An identical set can be used for the tracking frequency. However, it is recommended to use a lower push frequency to widen the push beam width compared with the track beam width to reduce the underestimation of tracked tissue displacement [31,32] so a lower frequency was used.

Changes in the voltage applied for the push will make the induced push less or more powerful creating shear waves of different amplitudes. The voltage applied was stepwise increased and a value of 40V was chosen for the ex vivo chicken liver and 28V for the hydrogel phantoms.

#### 2.3.1. Dispersion Velocity Calculation from Shear Wave Elastography Imaging (SWEI)

The measured shear wave velocity (SWV) can be used to determine tissue properties assuming a mechanical model of the tissue. For a linear, elastic, isotropic, homogeneous, and unbounded material, the SWV can be expressed in terms of the shear moduli μ and density ρ by the relation

(3) $$SWV\;=\;\sqrt{\mu /\rho }$$

The density of soft tissue is typically assumed to be 1000 $\mathrm{kg}/m^{3}$ and the SWV in units of m/s is equal to the square root of the shear moduli when it is expressed in units of kilopascals. In contrast, for a viscoelastic material, the shear moduli is a complex frequency-dependent quantity. Shear wave propagation in a viscoelastic material exhibits dispersion with a frequency-dependent phase velocity and shear attenuation [7].

The shear wave velocity dispersion curve was extracted from the ARFI using a phase difference method. First, the tissue velocity field was smoothed. This operation does not modify the phase velocity, only the amplitude, and the initial phase. The propagation of the plane wave in the sample along the x-direction is described by a 2D velocity field *v*(*x*,*t*). The phase ϕ(x,ω) of the wave at each frequency was obtained using a Fourier Transform of the tissue velocity field *v(x,t)*. For a monochromatic plane wave propagating in the direction *x*, the phase can be written as:

(4) ϕ(x,ω)=−Re[k(ω)]x

where k(ω) is the complex wavenumber and ω is the frequency. Thus, the shear wave phase velocity is:

(5) $$c_{s}\;=\;\frac{\omega }{-Re[k(\omega )]}$$

and the real part Re[k(ω)] of the wave number can be estimated from a linear fit of the phase ϕ(x,ω) along the propagation distance *x* [33,34,35].

Finally, dispersion curves are plots of shear wave velocity (SWV) as a function of angular frequency for ex vivo chicken liver samples and hydrogel phantoms.

#### 2.3.2. Tissue Motion Estimation

**Figure 6:**
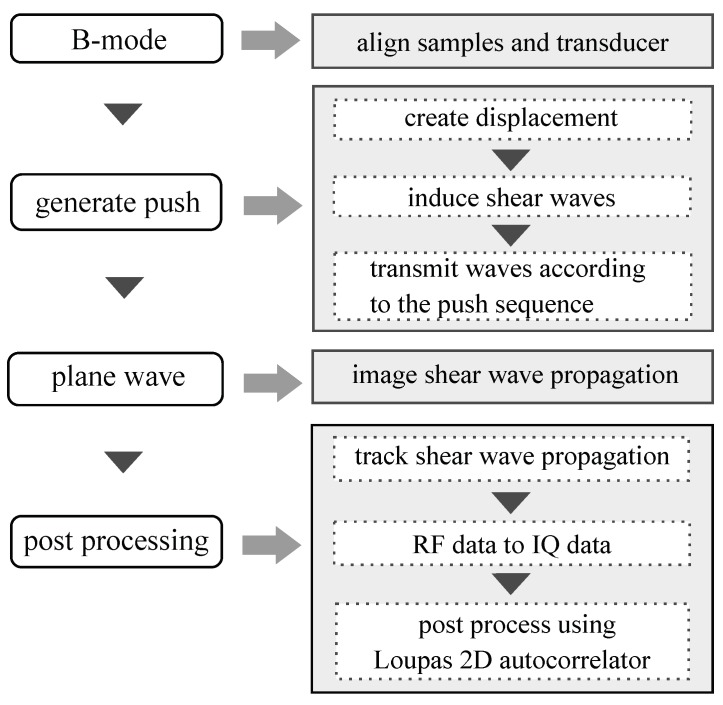
Procedure for tissue motion estimation using Shear Wave Elastography Imaging (SWEI) technique.

Tissue motion was determined using a phase-shift algorithm that operates on IQ data (in-phase and quadrature data). In this study, Loupas 2D auto-corrector algorithm was used to estimate the axial displacement caused by the propagation of the shear waves. The Loupas algorithm is an extension of the Kasai algorithm, which is used to post-process Verasonics data. It has the advantage of generating more accurate displacement estimations because it takes into account the center frequency [30,36]. Figure 6 shows a flow chart of how Verasonics generates SWEI and the steps needed to obtain the IQ data from an ARFI sequence. In this work, post-processing of the IQ data to obtain a displacement map was done using the Ultrasound Toolbox (USTB) [37].

## 3. Results

**Figure 7:**
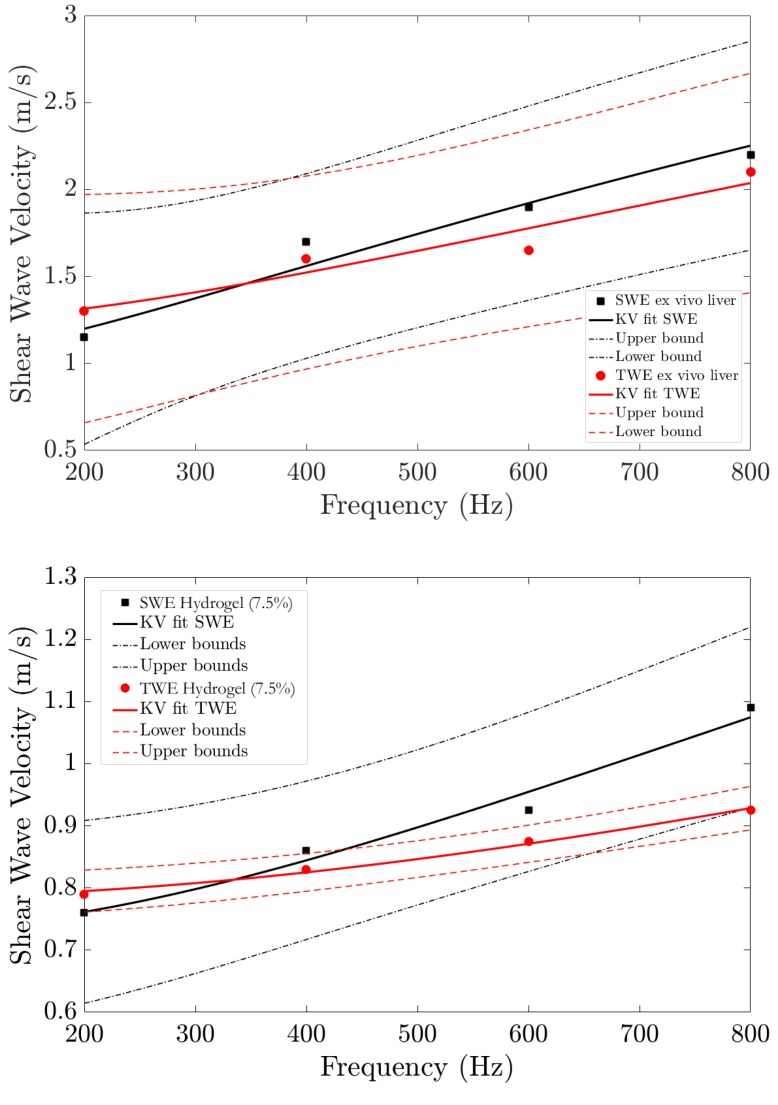
Dispersion curve for the two types of samples measured, square/circle marks are the values of shear wave velocity versus frequency via shear wave elastography imaging (SWEI) and torsional wave elastography (TWE) for ex vivo chicken liver samples (**top**) and hydrogel phantoms (**bottom**). Kelvin–Voigt (KV) fit is shown with solid lines in black color for SWEI and in red for TWE, and 95% confidence intervals are displayed with dashed lines.

The results of the scans are presented as mechanical biomarkers in terms of shear wave velocity, shear moduli, and viscosity. A comparison of shear wave velocity as a function of frequency for both TWE and SWEI techniques can be found in Figure 7. The sub-figure on the top is for fresh ex vivo liver samples and the one on the bottom is for hydrogel phantoms. Measurements were done within the frequency range of 200–800Hz. Solid lines are optimal fits of a Kelvin–Voigt rheological model.

The results show shear wave velocities go from 1.15 to 2.25m/s for SWEI and from 1.3 to 2.03m/s for TWE as mean values for the three liver samples. In the case of hydrogel phantoms, SWV values vary from 0.76 to 1.09m/s for SWEI and from 0.79 to 0.93m/s when scans were done via TWE. Both techniques show the same trend. These values are mean velocities for the two types of samples. ARFI based measurements were done three times in different liver areas. The results show a clear viscous trend in the samples. The results are in concordance with those presented in the literature [38,39,40].

**Figure 8:**
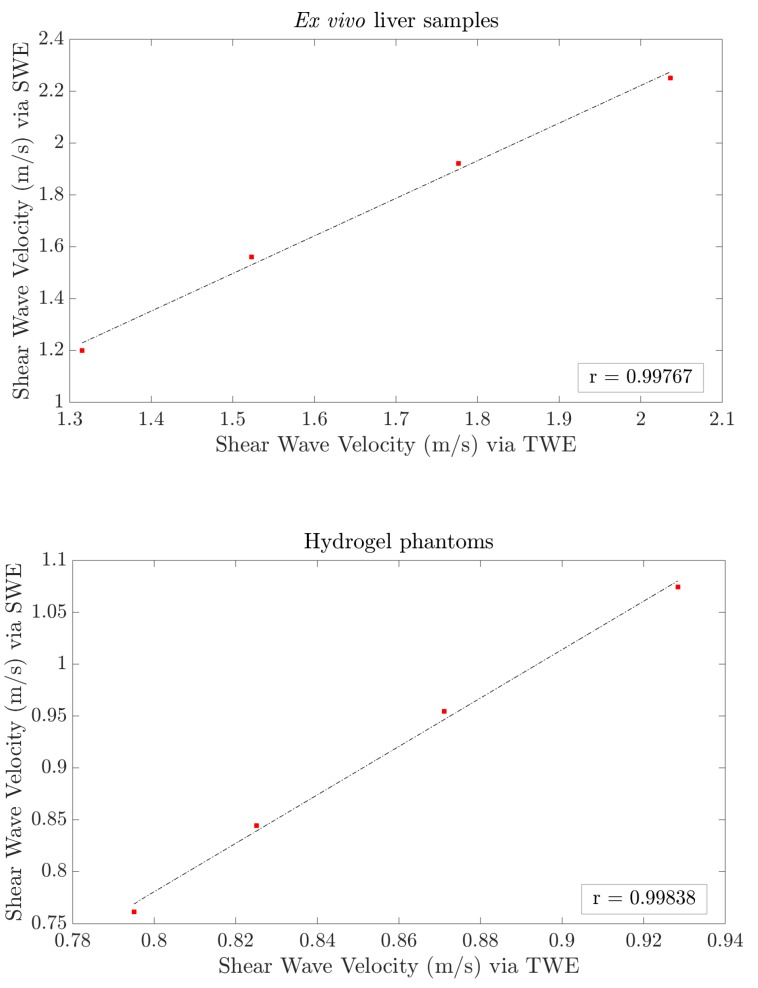
Pearson’s correlation between shear wave velocities via SWEI and TWE for both ex vivo liver samples (**top**) and hydrogel phantoms (**bottom**) at a frequency range from 200 to 800 Hz. Pearson correlation coefficients are 0.99767 for liver samples and 0.99838 for hydrogel phantoms.

A Pearson correlation coefficient was calculated to observe the degree of agreement between the reconstructed shear wave velocities obtained from both techniques, TWE and SWEI. The results are shown in Figure 8. A significant degree of agreement is observed, with a Pearson correlation coefficient of 0.99767 for liver samples and 0.99838 for hydrogel phantoms.

**Figure 9:**
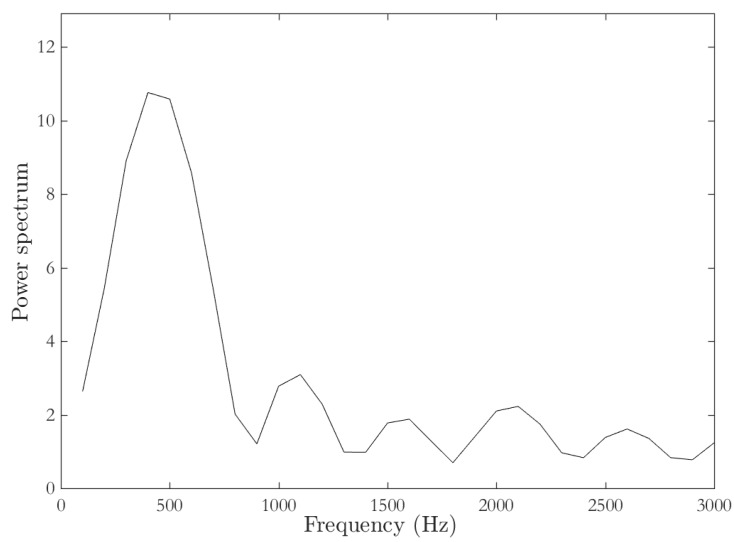
The power spectrum of the shear wave tracked by the 7.8Mhz (L11−5v) transducer for the ex vivo liver sample using a Verasonics vantage system.

Biomechanical elastic parameters obtained via TWE and SWEI in terms of shear moduli, μ, for both ex vivo chicken liver samples and hydrogel phantoms are tabulated in Table 4. Scans were made under a range of frequency from 200 to 800Hz. Measurements were done in this range of frequencies based on the power spectrum obtained from the shear wave tracked by the Verasonics transducer for liver samples, as shown in Figure 9. It can be observed that the energy concentration is within this range of frequencies (200–800Hz); frequencies above this range are considered noise.

Next, viscosity parameters for the same samples using two different rheological adjustments, namely Kelvin–Voigt and Maxwell, were determined (see Table 5). The results show the same trend; shear moduli are frequency dependent and increases with increasing frequency.

Verasonics Vantage systems measure and report shear wave velocity; therefore, to obtain the mechanical biomarkers in kPa, SWV values were transformed by Equation (3) to get shear moduli μ. In this study, it was assumed that tissue density is 1000 $\mathrm{kg}/m^{3}$.

**Figure 10:**
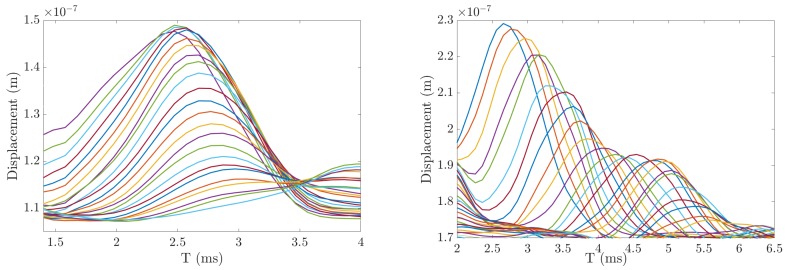
Experimental particle displacement versus time profiles at the focal depth resulting from the ARFI excitation. The ARFI moves the tissue in the axial and lateral position. In this figure, each displacement trace indicates a lateral position starting nearby the ARFI push focus to 24 lateral positions. Each individual color curve indicates the lateral position of a displacement trace for ex vivo liver sample II (**left**) and hydrogel phantom II (**right**). The curves show that, at farther distances (few milliseconds after the push), the particle displacement is reduced, since the shear wave dissipates.

Figure 10 shows particle displacement versus time profiles at 24 lateral positions for both an ex vivo liver sample and a hydrogel phantom.

**Figure 11:**
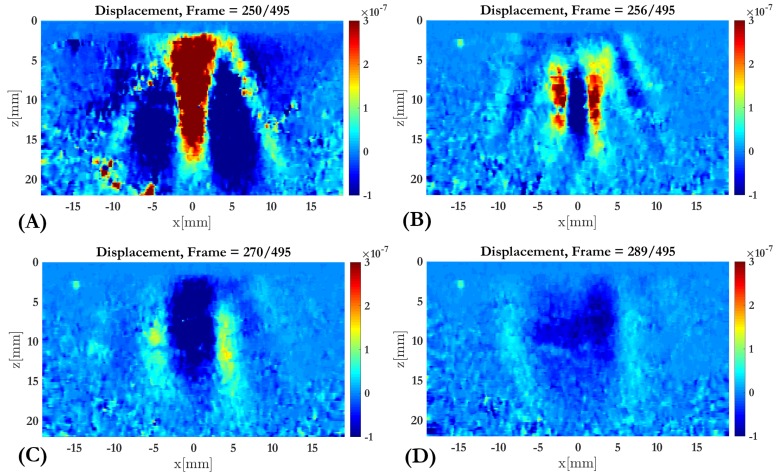
A sequence of displacement map (displacements are in meters) of ex vivo liver sample I due to ARFI excitation. The box represents the ROI (Region of Interest) chosen. The sequence from **A** to **D** show the push start (**A**) and the shear wave propagation in different frames (**A**–**D**) till its dissipation.

The axial displacement map obtained using the Loupas algorithm [36,37] after post-processing the IQ (in-phase and quadrature data) of ex vivo liver sample I is shown in Figure 11. We can see the ARFI push and the shear wave propagation. The y-axis represents the depth in mm of the scan, and the x-axis the lateral distance of the wave propagation. The sequence of the figure (from A to D) shows the localization of the ARF push and the shear wave lateral propagation away from the focus. Loupas 2D autocorrelator performs as the gold standard phase domain technique for motion estimation [41,42].

## 4. Discussion

The principal goal of this work was to be able to reliably quantify the mechanical properties of soft tissue using Torsional Wave Elastography (TWE). A combination of information from different techniques is required in order to improve our understanding of the tissue mechanical behavior. Knowing cut off values of the different emerging technologies and comparing them with technologies well known and working as the gold standard in the field of the elastography would originate a strong impact on clinical diagnoses. It is an ambitious goal, yet we have obtained promising results and the technique is being validated through this work and recent work of the group. Torsional wave elastography has been shown to be effective in obtaining biomechanical biomarkers [21,25,26,27,43].

In this work, SWEI was used to validate TWE, since it is the gold standard and one of the most important noninvasive techniques in quantifying the viscoelastic parameters [44,45]. A significant number of studies reinforce this decision; for instance, Kyoung et al. [46] showed that SWE is a good method to evaluate the usefulness of the stability index (SI) in liver stiffness measurements, demonstrating that this reduces the variability and increases the reliability in both free-breathing and breath-holding conditions. Samir et al. [47] estimated liver stiffness using SWE. The results obtained from the right upper lobe gave the best correlation with liver fibrosis severity and can potentially be used as a noninvasive test to differentiate intermediate degrees of liver fibrosis in patients with liver disease.

A validation study of five elastography techniques available commercially using individual tissue-mimicking liver fibrosis phantoms with different known Young’s moduli was performed by Mulabecirovic et al. [48]. They concluded that the SWE systems have very good repeatability and interobserver agreement. Dietrich et al. [49] presented guidelines and recommendations on the clinical use of liver ultrasound elastography; in their work, they firmly recommend comparison studies of all the technologies available to improve our knowledge on cut-off values for each system. Another comparison among commercially available techniques using SWE for the assessment of chronic liver diseases was presented by Friedrich-Rust et al. [50].

The reproducibility of the TWE technique was evaluated and found consistent with previous studies. The first validation of TWE was made by Callejas et al. [21] using a classical rheometer (limited to 50Hz), which is a quasi-static regime; the limitation of the previous work is that the measurements to obtain the shear moduli were made at frequencies well below the measurements made by TWE (300Hz to 2kHz). Therefore, in this work presented herein, the Verasonics Vantage system was used as the source to generate shear waves, allowing a comparison between the two methods in the same frequency range. The results are shown in Figure 7. The dispersion curves for the two types of samples measured, ex vivo liver samples and two tissue mimicking hydrogel phantoms, show the viscous response of the tissue. Each sample was measured several times by both techniques in different positions and under different pressures. Biological variability in the samples cannot be neglected; indeed, we observed different zones of rigidity in the same sample, which is true for all samples. Significant variability was found when different zones of the same sample were scanned by the same technique.

The values of shear wave velocities, from 1.15 to 2.25m/s for SWEI and from 1.3 to 2.03m/s for TWE as mean values for the three liver samples, agree with other results obtained from the literature [38,39,40]. However, the same figure shows that the curves representing TWE and SWEI results are spaced at high frequencies (>800Hz). This is probably because the attenuation is too high and the signal is dissipated. A similar observation was obtained from the hydrogel phantoms results. Pearson correlation coefficient shows good agreement between shear wave velocity (SWV) via TWE and SWEI, with values of 0.99767 for liver samples and 0.99838 for hydrogel phantoms, as reported in Figure 8.

Elastic biomarkers in terms of shear moduli in kPa under the frequency range of 200 to 800Hz show a good match between the techniques and report a similar tendency as SWV and therefore shear moduli are frequency dependent; increasing the frequency increases shear moduli values (Table 4).

One of the advantages of elasticity based images is that many soft tissues may share a similar capacity to reflect ultrasonic waves, but they may have different mechanical properties that can be used to visualize normal anatomy and trace pathological lesions more clearly. The liver is a viscoelastic structure, which is why changes in its viscosity would be closely related to liver diseases. Several authors suggest that changes in the transmission rate of mechanical vibration depend on the frequency [51,52,53]. Hence, SWE has some advantages over Transient Elastography (TE) [54,55]. Since Shear wave velocity is frequency dependent, it is possible to quantify the tissue viscosity from the shear wave dispersion curves [56,57,58,59]. In this study, viscoelastic biomarkers were obtained by fitting the model to the measured frequency. The results, as listed in Table 5, present significant differences between the two rheological models proposed, Kelvin–Voigt (KV) and Maxwell. The goodness of the adjustment shows that, in this case, KV model characterizes better both tissue samples and hydrogel phantoms. This opens up the debate to the elastography scientific community to present guidelines on which rheological model can express in the most concise way the characterization of soft tissue. Hydrogel phantoms show slightly better results than ex vivo liver samples, possibly for being more homogenous. Table 5 shows the parameters related to all the frequencies in the range of 200–800Hz, where the maximum energy is concentrated, and not all the frequencies shown in Figure 9. Frequencies above 800Hz are assumed to be noise.

TWE technique presents some advantages that make it interesting. First, it can reduce and isolate the spurious waves contamination (P-waves) [25,27]. Another advantage is its ability to accurately interrogate soft tissue mechanical functionality in cylindrical geometries. Dealing with this type of geometry is a challenge for current elastography approaches in small organs such as the uterine cervix, where SWE would generate bounces on the tissue walls and mask the signal received by the receiver. However, TWE technique generates less energy that does not generate rebounds [26]. Torsional waves propagate both radially and in depth, which is very advantageous in the case of multilayer tissue. The technique is able to characterize the different layers of the tissue when there is a clear difference between the stiffness of both, since shear waves propagate more quickly in stiffer media. The path of torsional waves from the transmitter to the receiver depends on the tissue scanned; the methodology for the characterization of bilayer tissue mimicking phantoms using TWE can be found in the work of Callejas et al. [22].

Finally, TWE technique deposits extremely low energy in the tissue, which makes it exceptionally safe. When the acoustic waves are used for fetal imaging, three parameters should be evaluated for safety considerations. These mechanical and thermal index parameters and their values for TWE can be found in the work of Callejas [60]. TWE technique has already been successfully applied in vivo in a recent work by Massó et al. [43] to determine uterine cervix elasticity in pregnant women.

In this work, the tissue was assumed to be isotropic, but when highly anisotropic tissue is scanned the assumptions made are not precise. Taking into account the fiber orientation, the anisotropy of the soft tissue is still a pending subject of commercial elastographic techniques, including TWE.

Future directions should include a study of the attenuation versus distance, as well as exploring different soft tissues and more complicated rheological models for more robust and accurate estimations of viscosity. Additionally, in vivo scans should continue to validate the TWE technique in organs where it presents an advantage over SWE. In summary, this work demonstrates that the proposed TWE technique has enough potential in the determination of mechanical properties of soft tissues. Preliminary results of ongoing work in vivo are encouraging.

## 5. Conclusions

In this work, the authors present a Torsional Wave Elastography (TWE) technique developed by part of our group to determine the mechanical properties of soft tissue. The results were compared with the ones obtained from a commercial SWEI alternative. A programmable SWE-system for ex vivo samples was implemented and evaluated. The results of shear wave velocities and shear moduli for both ex vivo and hydrogel phantoms are in concordance with the literature. At the moment, we strongly believe that these results are promising and can be considered as a baseline for future studies on TWE. The objective was reached and TWE has been shown to be able to capture the tissue variability with respect to the frequency with a tendency close enough to the gold standard in elastography. Exploring the TWE technique in other soft tissues will be interesting future work, as will the study of attenuation versus time in in vivo measurements.

## Figures and Tables

**Table 1:** Torsional wave elastography (TWE) technique acquisition parameters for both ex vivo liver samples and hydrogel phantoms.

Measurements Acquisition Parameters	Value
Sampling frequency	80 Hz (Decimated 10 × after 800 Hz)
Ring-disc radius	3 mm
Frequency	200–800 Hz
Averaging	10 ×
Excitation power	20 V

**Table 2:** Properties of the L11−5v Verasonics transducer.

Property	Value
Number of Elements	128
Pitch (mm)	0.3
Elevation focus (mm)	18
Sensitivity (dB)	−52 ± 3

**Table 3:** SWEI acquisition parameters for L11−5v Verasonics transducer.

Parameter	Value for the L11−5v Transducer
Push frequency (MHz)	4.8
Track frequency (MHz)	7.81
Push duration (cycles)	1000
Pulse repetition interval (μs)	100
Impulse duration (cycles/(μs))	1000, 128
Impulse focus (mm)	16 for ex vivo liver and 12 for hydrogel phantoms
Beam focus configuration	Plane wave, fully open
IQ data beam forming sampling frequency	0.25λ
Excitation voltage (V)	40 for ex vivo liver and 28 for hydrogel phantoms
Sampling frequency(Hz)	3000
Number of transmission channels	128
Number of reception channels	128

**Table 4:** Shear moduli in kPa for both ex vivo liver samples and hydrogel phantoms obtained from torsional wave elastography (TWE) and shear wave elastography imaging (SWEI) techniques.

	Elastic Parameter: Shear Moduli in kPa			
	Ex Vivo Liver Samples		Hydrogel Phantoms	
**Frequency (Hz)**	$\mu_{\mathrm{TWE}}$	$\mu_{\mathrm{SWEI}}$	$\mu_{\mathrm{TWE}}$	$\mu_{\mathrm{SWEI}}$
200	1.69±0.78	1.32	0.62±0.04	0.58
400	2.66±0.23	2.82	0.68±0.05	0.74
600	2.69±0.47	3.69	0.78±0.065	0.85
800	4.00±0.42	4.84	0.86±0.055	1.16

**Table 5:** Viscoelastic parameters for ex vivo liver samples and hydrogel phantoms obtained from torsional wave elastography (TWE) and shear wave elastography imaging (SWEI) techniques.

Sample	Fit	Viscous Parameters				The Goodness of Fit R-square	
		TWE		SWEI		TWE	SWEI
Ex vivo liver	Kelvin–Voigt (KV)	μ=1.512 kPa	η=0.536 Pa·s	μ=1.019 kPa	η=0.628 Pa·s	0.9198	0.9572
	Maxwell (M)	$\mu_{1}\;=\;5.773$ kPa	$\mu_{2}\;=\;4.316$ Pa·s	$\mu_{1}\;=\;13.720$ kPa	$\mu_{2}\;=\;3.712$ Pa·s	0.835	0.9861
Hydrogel phantom	Kelvin–Voigt (KV)	μ=0.615 kPa	η=0.093 Pa·s	μ=0.532 kPa	η=0.148 Pa·s	0.9926	0.9764
	Maxwell (M)	$\mu_{1}\;=\;0.827$ kPa	$\mu_{2}\;=\;2.897$ Pa·s	$\mu_{1}\;=\;1.267$ kPa	$\mu_{2}\;=\;1.663$ Pa·s	0.7879	0.8237

## Abbreviations

The following abbreviations are used in this manuscript:

TWE	Torsional Wave Elastography
SWEI	Shear Wave Elastography Imaging
DE	Dynamic Elastography
ARFI	Acoustic Radiation Force Impulse
MRE	Magmatic Resonance Elastography
SWE	Shear Wave Elastography
KV	Kelvin–Voigt
M	Maxwell
PIP	Probabilistic Inverse Problem
FDTD	Finite Difference Time Domain
TOF	Time of Flight
ROI	Region of Interest
US	Ultrasonics
IQ	In-phase and Quadrature Data
CNC	Computer Numerical Control
SWV	Shear Wave Velocity
